# An evaluation of POSSUM and P-POSSUM scoring in predicting post-operative mortality in a level 1 critical care setting

**DOI:** 10.1186/1471-2253-14-104

**Published:** 2014-11-18

**Authors:** Sarah Scott, Jonathan N Lund, Stuart Gold, Richard Elliott, Mair Vater, Mallicka P Chakrabarty, Thomas P Heinink, John P Williams

**Affiliations:** Division of Medical Sciences and Graduate Entry Medicine, School of Medicine, University of Nottingham, Royal Derby Hospital, Derby, DE22 3DT UK; Department of Anesthesia and Critical Care, Royal Derby Hospital, Derby, DE22 3NE UK; MRC/Arthritis Research UK Centre for Musculoskeletal Ageing Research, University of Nottingham, Nottingham, NG7 2UH UK

## Abstract

**Background:**

POSSUM and P-POSSUM are used in the assessment of outcomes in surgical patients. Neither scoring systems’ accuracy has been established where a level 1 critical care facility (level 1 care ward) is available for perioperative care. We compared POSSUM and P-POSSUM predicted with observed mortality on a level 1 care ward.

**Methods:**

A prospective, observational study was performed between May 2000 and June 2008. POSSUM and P-POSSUM scores were calculated for all postoperative patients who were admitted to the level 1 care ward. Data for post-operative mortality were obtained from hospital records for 2552 episodes of patient care. Observed vs expected mortality was compared using receiver operating characteristic (ROC) curves and the goodness of fit assessed using the Hosmer-Lemeshow equation.

**Results:**

ROC curves show good discriminative ability between survivors and non-survivors for POSSUM and P-POSSUM. Physiological score had far higher discrimination than operative score. Both models showed poor calibration and poor goodness of fit (Hosmer-Lemeshow). Observed to expected (O:E) mortality ratio for POSSUM and P-POSSUM indicated significantly fewer than expected deaths in all deciles of risk.

**Conclusions:**

Our data suggest a 30-60% reduction in O:E mortality. We suggest that the use of POSSUM models to predict mortality in patients admitted to level 1 care ward is inappropriate or that a recalibration of POSSUM is required to make it useful in a level 1 care ward setting.

## Background

Despite advances in surgical technique and peri-operative care, high-risk surgical procedures still carry a significant risk, with approximately 20,000 – 25,000 deaths per year (1.6% of all surgical interventions) following a surgery in the UK [1]. In an attempt to quantify the risk of peri-operative morbidity and mortality, a number of scoring systems have been developed [2]. The American Society of Anesthesiologists Physical Status score (ASA-PS) is perhaps the best known, using a subjective assessment of physical ability to categorize patients into one of five groups. ASA-PS score shows good correlation with postoperative outcome for patient populations in a number of surgical settings, but does not describe individual patient risk and cannot account for surgical procedure, preoperative optimisation or individual differences in postoperative care setting. Consequently ASA-PS shows a poor ability to identify individuals likely to experience complications in the postoperative period [2]. To address some of these limitations Copeland et al. [3] developed a scoring system that they hoped could easily be used to help provide both retrospective and prospective analysis of the risk of post-surgical mortality and morbidity. This scoring system was named POSSUM (Physiological and Operative Severity Score for the enUmeration of Mortality and morbidity). With its derivations is now the most widely validated predictive scoring systems used in perioperative care [2].

In order to assess post-operative risk, POSSUM was originally developed as a tool to compare morbidity and mortality in a wide range of general surgical procedures in order to facilitate surgical audit and to compare the performance of individual units. It was intended that the risk of a surgical procedure could be calculated based on a patient’s physiological condition and then pooled, thus allowing a more accurate comparison of a unit’s (or individuals’) performance. In 1998 researchers identified a need to adjust the logistic regression analysis used in POSSUM scoring to better predict mortality [4]. This resulted in a second scoring system using the same standard data set, Portsmouth-POSSUM (P-POSSUM). Both of these scoring systems are widely used in the UK helping guide better utilization of health care resources for postoperative patients.

The POSSUM score describes 18 factors in two component parts; 12 physiological factors (PS) and 6 operative factors (OS). Each factor is scored exponentially increasing from 1 to 8 (1, 2, 4, 8) dependent upon grading. From these values predicted mortality can be calculated using formulae described. Although P-POSSUM, the refinement of the original scoring system, collects the same 18 physiological and operative parameters, a different calculation formula is employed to determine predicted mortality.

The Comprehensive Critical Care report [5] published in 2000 by the UK’s Department of Health Expert Group defined four levels of patient care which hospitals should provide to meet the needs of each individual patient.

- Level 0: Patients whose needs can be met through normal ward care in an acute hospital.
- Level 1: Patients at risk of their condition deteriorating, or those recently relocated from higher levels of care, whose needs can be met on an acute ward with additional advice and support from the critical care team.
- Level 2: Patients requiring more detailed observation or intervention including support for a single failing organ system or post-operative care and those ‘stepping down’ from higher levels of care.
- Level 3: Patients requiring advanced respiratory support alone or basic respiratory support together with support of at least two organ systems. This level includes all complex patients requiring support for multi-organ failure.

Many postoperative surgical patients require higher levels of care than level 0, and while most acute hospitals have High Dependency and Intensive Care (level 2 and 3) facilities, few have level 1 care environments. This type of environment was developed to bridge the gap in care between levels 2 and 3 care and level 0 (general ward) care.

While many previous investigators have demonstrated the validity of POSSUM and P-POSSUM in a variety of surgical disciplines [6–13], and other smaller studies have addressed the relationship between POSSUM/P-POSSUM predicted mortality and observed mortality of level 2 and 3 care [3, 14, 15], none have established these scoring systems’ accuracy where a level 1 critical care facility (level 1 care ward) is available for perioperative care.

Additionally although POSSUM and P-POSSUM scoring systems have been validated for a number of surgical specialties, they are now 23 and 16 years old respectively and may not accurately reflect the risk faced by the today’s surgical patient admitted to a level 1 care ward in the UK. The aim of our study was to compare POSSUM and P-POSSUM predicted mortality with observed mortality on a level 1 care ward. Post-operative morbidity was not assessed.

## Methods

Ethics: Ethical approval for this study (10/H0405/79) was provided from the Research Ethics Committee at Derby Hospitals NHS Foundation Trust, Derby, UK (chairperson Mr Peter Korczak) on 2 September 2010.

Anonymized data for all surgical patients over the age of 18 years admitted postoperatively to the 16-bed level one care ward at our institution between May 2000 and June 2008 was collected prospectively. The level 1 care ward, positioned directly opposite the intensive care unit, provided invasive arterial and central venous pressure monitoring, 24-hour care and resident physician cover, and consultant anesthetist and surgical team joint care during daytime each day, although there were no facilities for organ support beyond the use of low-dose vasopressor infusions to correct epidural-associated hypotension. The nursing staff worked a full shift pattern with one trained nurse to every 2.5 patients. Patients were admitted to the level one care ward after gastrointestinal, urological or gynaecological surgery.

PS and OS were calculated for each admitted patient and entered onto a dedicated database by the admitting physician, and from these values POSSUM and P-POSSUM scores were calculated for each patient. POSSUM and P-POSSUM mortality formulae are shown below:

$$\begin{array}{lll} \mathrm{POSSUM} & Ln\left[R/\left(1\;-R\right)\right]= & -7•04\;+\left(0•13\;\times \;PS\right)\\ & & +\left(0•16\;\times \;\mathrm{OS}\right)\\ P-\mathrm{POSSUM} & Ln\left[R/\left(1\;-R\right)\right]= & -9•065+\left(0•1692\times PS\right)\\ & & +\left(0•155\;\times \;\mathrm{OS}\right) \end{array}$$

In this study hospital mortality was obtained from hospital mortality records with mortality beyond 30 days not considered significant. Individuals with incomplete data or not directly admitted to the level 1 care ward were excluded from analysis.

### Statistical analysis

POSSUM and P-POSSUM mortality prediction models were assessed by measuring the ability of the models to discriminate between patients who died and those who did not, observed over expected mortality ratios and calibration fit of the models across various risk bandings.

One of the most common measures of test discrimination is the receiver operating characteristic curve (ROC), a plot of sensitivity *vs* 1 - specificity. This curve assesses how well a test or model discriminates individuals into two classes, such as diseased and non-diseased comparing the test against the actual outcome. Discrimination is assessed by measuring the area under the curve (AUC) of the plot of sensitivity *vs* 1 – specificity for all test cut off points. The AUC is also known as the C-statistic or C-index, with 1 being a perfect discriminating test and 0.5 having no discriminating value [16–19]. Discrimination is acceptable for 0.7 ≤ AUC <0.8 good to excellent for 0.8 ≤ AUC < 0.9 and excellent for AUC ≥0.9 [18]. Analysis via ROC curves therefore allows not only for test discrimination to be judged, but also for different diagnostic tests to be compared.

Model performance should be assessed not only by ability to discriminate between diseased and non-diseased individuals, but also by ability to assess whether or not observed events match expected events over the range of the model, for this calibration between observed and predicted risk needs to be assessed. The Hosmer-Lemeshow equation is a commonly used goodness of fit test which compares observed outcome to predicted outcome within bands of risk. Risk bands can be divided equally into deciles of risk, as demonstrated in Tables 1 and 2, or to give equal values of predicted deaths per band (ideally where that number is ≥5). Large χ^2^_HL_ values suggest poor fit, with calibration considered to be poor if p ≤0.05. Observed to expected mortality ratios were recorded for both models, with expected mortality the mean of the expected mortalities in each decile of risk calculated from the model (POSSUM or P-POSSUM).

**Table 1:** **Hosmer-Lemeshow goodness of fit test for POSSUM for 30-day mortality**

Deciles of risk (%)	Number of patients	Number of observed deaths	Number of expected deaths	Mean risk of predicted mortality	O:E (95% CI)	X ^2^HL statistic
0-10	1501	15	71.96	0.05	0.21 (0.12 – 0.36)	47.36
10-20	514	19	72.88	0.14	0.26 (0.16 – 0.43)	46.42
20-30	216	16	52.36	0.24	0.31 (0.17 – 0.53)	33.33
30-40	133	9	45.64	0.34	0.20 (0.10 – 0.40)	44.78
40-50	66	6	29.38	0.45	0.20 (0.09 – 0.49)	33.54
50-60	51	8	27.61	0.54	0.29 (0.14 – 0.64)	30.37
60-70	34	5	21.84	0.64	0.23 (0.09 – 0.61)	36.29
70-80	22	7	16.40	0.75	0.43 (0.18 – 1.04)	21.26
80-90	12	2	10.00	0.83	0.20 (0.05 – 0.81)	38.40
90-100	3	1	2.87	0.96	0.35 (0.07 – 3.82)	27.08
0-100	2552	88	350.94		0.25 (0.20 – 0.32)	**358.73**

X^2^HL statistic =358.73; df =8; p <0.0001.

**Table 2:** **Hosmer-Lemeshow goodness of fit test for P-POSSUM for 30-day mortality**

Deciles of risk (%)	Number of patients	Number of observed deaths	Number of expected deaths	Mean risk of predicted mortality	O:E (95% CI)	X ^2^HL statistic
0-10	2150	38	52.42	0.02	0.73 (0.69-0.76)	4.06
10-20	203	19	28.64	0.14	0.66 (0.61-0.72)	3.78
20-30	73	7	18.27	0.25	0.38 (0.32-0.45)	9.27
30-40	46	6	16.05	0.35	0.37 (0.30-0.45)	9.67
40-50	29	6	13.14	0.45	0.46 (0.38-0.54)	7.10
50-60	17	3	9.36	0.55	0.32 (0.23-0.42)	9.63
60-70	18	5	11.45	0.64	0.43 (0.35-0.53)	10.01
70-80	10	2	7.62	0.76	0.26 (0.17-0.37)	17.48
80-90	3	1	2.53	0.84	0.40 (0.23-0.59)	6.05
90-100	3	1	2.87	0.96	0.40 (0.23-0.59)	29.62
0-100	2552	88	162.35	0.06	0.54 (0.47-0.62)	**106.67**

X^2^HL statistic =106.67; df =8; p <0.0001.

## Results

In total 3741 patient episodes were analyzed, with 1189 patients excluded due to insufficient data (n = 690), or because individuals were not directly admitted to the unit following surgery (n = 499). Of the remaining 2552 patient episodes included in analysis, 88 died by the 30^th^ post-operative day, an overall mortality rate of 3.45%. The mean age of individuals studied was 62.58 ^+^/- 15.68 SD, with 52.5% being male. Demographic data is shown in Table 3.

**Table 3:** **Demographics of level one care patients analysed with length of stay on the level 1 care ward**

	Total (%)	Died (%)	Length of stay on unit, days (Median ^+^/- IQR)
**General surgery**			
Elective	969 (67.4%)	27 (2.8%)	2 (2–3)
Emergency	469 (32.6%)	46 (9.8%)	2 (1–3)
Male	756 (52.3%)	38 (5.0%)	2 (1–3)
Female	682 (47.7%)	35 (5.1%)	2 (1–3)
Total	1438 (56.3%)	73 (5.1%)	2 (1–3)
**Urology**			
Elective	723 (89.4%)	3 (0.4%)	3 (2–4)
Emergency	86 (10.6%)	5 (5.8%)	2 (1–3)
Male	575 (71.1%)	8 (1.4%)	2 (1–3)
Female	234 (28.9%)	3 (1.3%)	3 (2–4)
Total	809 (31.7%)	11 (1.4%)	2 (2–4)
**Gynaecology**			
Elective	232 (77.8%)	3 (1.3%)	2 (1–2)
Emergency	66 (22.1%)	0 (0.0%)	1 (1–2)
Male	0 (0.0%)	0 (0.0%)	0
Female	298 (100%)	3 (100%)	2 (1–2)
Total	298 (11.7%)	3 (1.0%)	2 (1–2)
**Other**			
Elective	1 (14.3%)	0 (0.0%)	1
Emergency	6 (85.7%)	1 (16.6%)	1 (0.75-2)
Male	4 (57.1%)	1 (25.0%)	1.5 (1–2)
Female	3 (42.9%)	0 (0.0%)	1 (0–1)
Total	7 (0.7%)	1 (14.3%)	1 (1–2)
**All surgery**			
Elective	1926 (75.5%)	33 (1.7%)	2 (2–3)
Emergency	626 (24.5%)	55 (8.7%)	2 (1–3)
Male	1340 (52.5%)	47 (3.5%)	2 (1–3)
Female	1088 (42.6%)	41 (3.8%)	2 (1–3)
Total	2552 (100%)	88 (3.4%)	2 (1–3)

**Figure 1:**
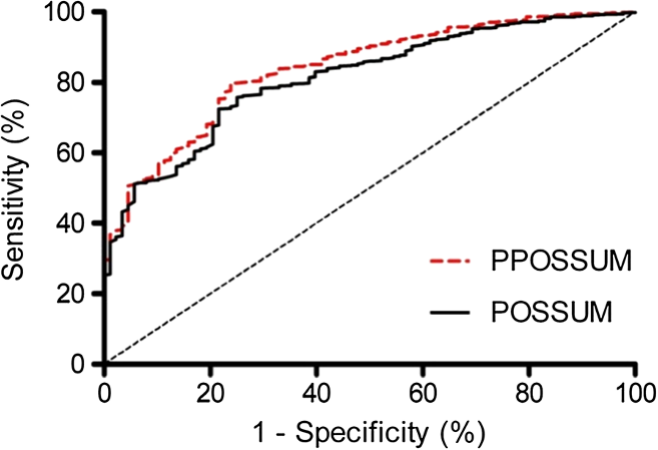
**Receiver operator characteristic curve for performance of POSSUM and P-POSSUM.**

**Figure 2:**
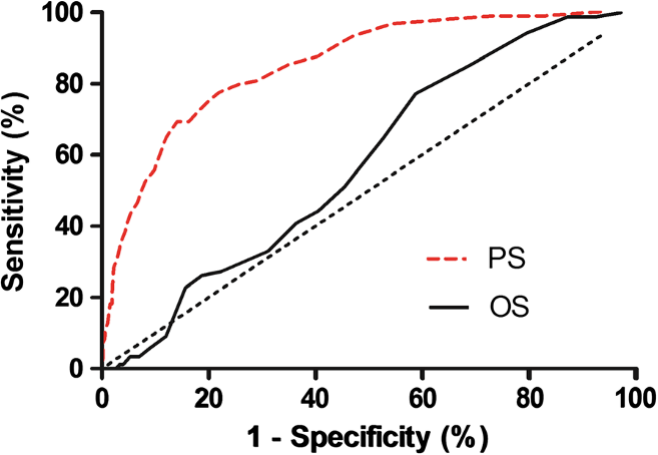
**Receiver operator characteristic curve for performance of physiological score and operative score alone.**

Analysis of ROC curves show good discriminative ability between survivors and non-survivors for both POSSUM and P-POSSUM models in the level 1 care ward setting when data from all patients admitted to the level 1 care facility are analyzed (Figure 1). (In this example discrimination is the ability to choose a random patient pair, with the highest score belonging to the patient in the random pair who does not survive). Area under the curve for the POSSUM receiver operator curve (AUC POSSUM) was 0.81 ± 0.02 SE, and 0.84 ± 0.02 SE for P-POSSUM (Figure 1). Physiological score alone was also found to have far higher discrimination than operative score alone; AUC physiological score was 0.85 ± 0.02 SE and 0.58 ± 0.03 SE for operative score (Figure 2).

**Figure 3:**
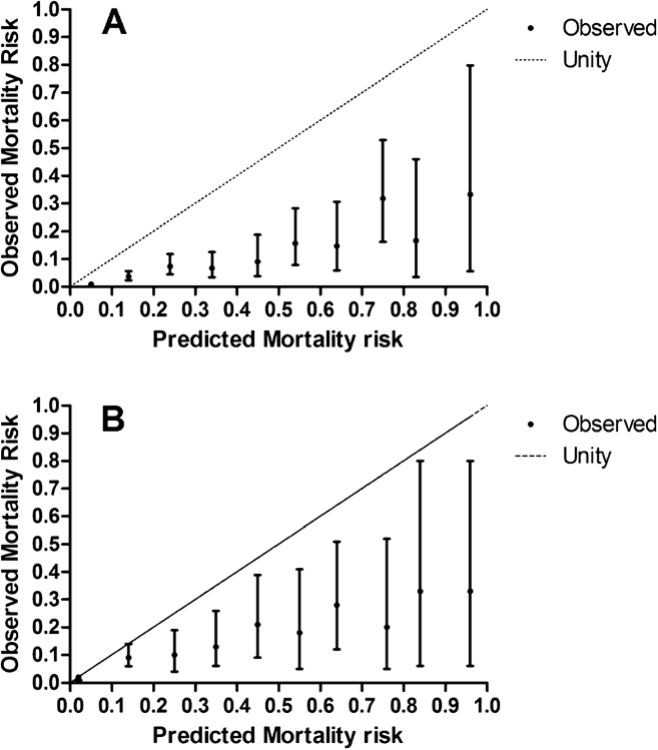
**Calibration curves for observed mortality, with 95% confidence intervals, showing poor fit of predicted mortality compared to observed mortality across all risk deciles.** Perfect test shown by line of unity. **A** - POSSUM. **B** - P-POSSUM.

Despite both models showing good discrimination when assessed via ROC curve, Hosmer-Lemeshow testing showed poor calibration assessed by goodness of fit for both POSSUM and P-POSSUM models for all patients admitted. This lack of fit was evident for both models, with both demonstrating large χ^2^_HL_ values (POSSUM: χ^2^_HL_ statistic = 358.73; df = 8; p <0.0001; P-POSSUM: χ^2^_HL_ statistic = 106.67; df = 8; p <0.0001) (Tables 1 to 2). This indicates that both POSSUM and P-POSSUM models are poor predictors of mortality when deciles are divided according to equal predicted risk, or equal risk ranges (Figure 3). The χ^2^_HL_ statistic for POSSUM and P-POSSUM with 4 bands of risk to ensure clinically meaningful risk bands with equal expected mortalities within each risk band (performed as described by Prytherch et al. [4]), also yielded large χ^2^_HL_ values for POSSUM and P-POSSUM of 307.78 and 75.66 respectively. This again indicated that the models displayed poor fit even when the test was optimized (p <0.0001). Observed to expected (O:E) mortality ratio calculated for POSSUM and P-POSSUM indicated significantly fewer than expected deaths in all deciles of risk for both models, with overall O:E ratios of 0.25 (0.20-0.32 CI) for POSSUM and 0.54 (0.47-0.62 CI) for P-POSSUM. Hosmer-Lemeshow testing also showed poor calibration assessed by poor goodness of fit for individual surgical specialties with both POSSUM and PPOSSUM demonstrating large χ^2^_HL_ values for all specialties (**GENERAL SURGERY**: POSSUM: χ^2^_HL_ statistic = 199.9; df = 8; p <0.001; P-POSSUM: χ^2^_HL_ statistic = 79.35; df = 8; p <0.001. **UROLOGY**: POSSUM: χ^2^_HL_ statistic = 114.2; df = 8; p <0.001; P-POSSUM: χ^2^_HL_ statistic = 26.59; df = 8; p <0.05. **GYNAECOLOGY**: POSSUM: χ^2^_HL_ statistic = 53.0; df = 8; p <0.001; P-POSSUM: χ^2^_HL_ statistic = 19.6; df = 8; p <0.05).

Given this poor fit, logistic regression analysis was performed on the data set using IBM SPPS Statistics 19 (IBM, New York, USA). PS and OS were used as continuous predictor variables, urgency of surgery as a categorical predictor, and mortality as the categorical dependent variable (Table 4). From this the following predictor equation was derived for mortality employing the originally recorded operative and physiological scores and urgency of surgery, and termed S-POSSUM: Ln[R/(1 − R)] = −6.505 + (0.144 × PS) + (0.03 × OS) + (1.057 × NE), where R is the risk of mortality and NE = 1 for non-elective surgery and 0 for elective surgery. Hosmer Lemeshow testing of this model showed no significant lack of fit (χ^2^_HL_ statistic = 4.503; df = 8; p = 0.81).

**Table 4:** **Co-efficients used in logistic regression analysis**

					95% C.I. for Exp (B)	
	B	S.E.	P	Exp (B)	Lower	Upper
PScore	.144	.014	.000	1.155	1.124	1.187
OScore	.030	.021	.153	1.031	.989	1.075
Emergency	−1.057	.244	.000	.348	.215	.561
Constant	−6.505	.557	.000	.001		

## Discussion

In this, the largest study to date in this group of patients, we found that P-POSSUM and POSSUM scoring models made good to excellent discrimination between survivors and non-survivors in a range of surgical specialties treated on a level 1 care ward post-operatively, but with poor calibration across risk bands and less mortality at all deciles of risk than predicted by both models. Overall observed to expected mortality ratios were significantly lower than predicted for POSSUM and P-POSSUM, with O:E mortality 0.25 (0.20-0.32 CI) for POSSUM and 0.54 (0.47-0.62 CI) for P-POSSUM. These mortality rates are not only better than standardized mortality ratios observed for general surgical patients treated in a variety of postoperative environments, but are also comparable to the limited literature documenting POSSUM calculated O:E mortality in level 2 and 3 care areas for our patient population [4, 5, 11]. These results suggest that either POSSUM scoring requires further recalibration, that level 1 care ward saves an extra 50% of lives at risk after operation, or that the scoring systems themselves are at fault. Regardless of reason, POSSUM scoring provided poor prediction of risk in the level 1 care ward setting.

Previous studies concentrating on surgical specialty alone [4, 20–22] have observed significant variation from the predicted models at particular deciles of risk banding, most notably the lower bands of predicted risk. Investigators have also explored the performance of these models for a variety of surgical specialties, with a systematic review [23] reporting a mean O:E of 0.9 for P-POSSUM (confidence interval 0.88-0.92) in colorectal cancer, and a further large multi-centre study describing a mean O:E mortality of 1.0 (CI 0.88-1.13) [21]. However, discrepancy between observed to expected mortality amongst individual studies is large. Similar O:E discrepancies have been reported in other surgical specialties [24, 25].

With other scoring systems frequently employed in critical care areas, research describing the appropriateness of POSSUM based models to predict mortality in areas delivering higher dependency care is sparse. Cavaliere [14] and Organ [8] report surgical intensive care unit observed mortalities of approximately half that predicted by POSSUM and P-POSSUM scoring, while Clarke [15] found higher O:E mortality in a small number of patients undergoing emergency laparotomy admitted to a post-anesthesia care unit (PACU)-ward (O:E 0.82), compared to a PACU-HDU-ward pathway or ICU-high dependency unit (HDU)-ward pathway (O:E 0.0; O:E 0.69, respectively). Level 2 and 3 care is currently not thought appropriate for the majority of post-operative patients, however targeted critical care admission for pre-identified high-risk surgical patients may demonstrate improved outcome [26–28]. Demonstration of a comparable or improved patient outcome following level 1 care in comparison to higher more intensive levels of care postoperatively for individuals with lower risk could have obvious economic implications.

While evidence suggests that 30 day mortality following surgery in various surgical specialties has not changed over the last 20 years, this present study indicates that level 1 postoperative care significantly outperforms POSSUM prediction models, is comparable to level 2 and 3 care in this patient group and is superior to surgical specialty predicted mortality. Moreover interest has recently focused on the importance of physiological assessment and urgency of surgery as predictors of perioperative mortality [29]. Logistic regression analysis of data from this present study supports this view, suggesting that more selective assessment of patients destined for a level 1 care environment may be possible by placing greater weight on physiological score and urgency rather than nature of operation and by utilizing our proposed scoring system S-POSSUM. Taken together these findings indicate that further research is required in the area to better quantify the extent to which physiological score and urgency of surgery influence outcome. In addition further prospective testing needs to be undertaken to assess the effectiveness of any novel scoring system.

We acknowledge that there are limitations in our study. Firstly individuals admitted to the level 1 care ward from level 2 or 3 wards and those with inadequate data collection were excluded from analysis. We feel that this exclusion of data was justified given our intention to specifically study the effect of level 1 care and not the effect of prior level 2/3 medical care. It is unlikely that the excluded patients differed significantly from those included, as further analysis demonstrates a similar demographic profile and length of stay. Secondly, with such a large data set there is the potential for the original source data to have been incorrectly documented. Given the contemporaneous recording and simplicity of scoring systems used we feel any error within such a data set to be minimal. Thirdly, individuals may have left the region prior to death within 30 days of surgery and therefore failed to be collected as mortality statistics; we believe that this is unlikely, and this is in line with previous POSSUM analyses [3, 4]. Fourthly patients admitted to the level one care ward, discharged, and then readmitted to level 1 care following a second surgery represent two data entries. Whilst the anonymous nature of the data makes it difficult to accurately quantify the number of readmissions, data analysis using demographic data (date of birth, gender) suggests a readmission rate of around 1%.

Next, although this is the largest study to date in this population of patients, in terms of absolute number of patients included, the number of deaths remains relatively small. Expanding the study to include other institutions may improve the power of the study, but was not logistically feasible. Likewise, this is single center study and the apparent poor fit of both POSSUM and P-POSSUM models may simply be due to our unit performing well, rather than a problem with the model per se. Certainly, further validation in other centers is required prior to our proposed recalibration of POSSUM entering routine clinical usage. The data set analyzed spans an eight-year time period during which medical care may well have progressed. Subdivision of this period into temporal quintiles showed no significant change in POSSUM scoring or O:E mortality. However the manner of our analysis of these records provides us with a data set of significant size and obliges us to draw our conclusions across the whole of this span as an entirety.

Finally, it is important to remember that all risk prediction models, including POSSUM and P-POSSUM, lack specificity for individual patients, they merely suggest how frequently an outcome occurs at a population level. By their nature, risk prediction models require dichotomous decisions to be made about the presence or absence of specific co-morbidities and they cannot take into account subtleties in diseases, which usually occur as a continuum [30]. Consequently caution must be exercised when applying these models to individuals.

## Conclusion

This is the largest study to date examining O:E mortality in a level 1 care ward. Our data suggest a 30-60% reduction in observed mortality over that predicted by POSSUM or P-POSSUM. We suggest that the use of POSSUM models to predict mortality in patients admitted to level 1 care ward is inaccurate and propose a recalibration of POSSUM (S-POSSUM: Ln[R/(1 − R)] = −6.505 + (0.144 × PS) + (0.03 × OS) + (1.057 × NE)) for use in a level 1 care ward setting.

## Abbreviations

ASA-PS: American Society of Anesthesiologists Physical Status
(P)-POSSUM: (Portsmouth)-physiological and operative severity score for the enumeration of mortality and morbidity
PS: Physiological factors
OS: Operative factors
ROC: Receiver operating characteristic
AUC: Area under the curve
O: E: Observed to expected
PACU: Post anesthesia care unit
HDU: High dependency unit
ICU: Intensive care unit.
